# Preliminary report: The most considerable outbreak of Legionnaires' disease in France in the last two decades, Albertville, September 2025

**DOI:** 10.2807/1560-7917.ES.2025.30.45.2500813

**Published:** 2025-11-13

**Authors:** Emmanuelle Vaissière, Audrey Merlet, Muriel Deher, Matthieu Curtil-Dit-Galin, Jean-Marc Yvon, Albane Beaupoil, Emmanuel Forestier, Jean-Marie Kuntzelmann, Brune Joannard, Aymeric Provost, Guillaume Spaccaferri, Christelle Nolibos, Laetitia Beraud, Nathalie Grangeret, Christine Campese, Sophie Jarraud, the Albertville LD Outbreak Team Investigation, The Albertville LD Outbreak Team Investigation, Léo Blervaque, Elise Brottet, Marie-Christine Carret, Florence Culoma, Erica Fougère, Olivier Gaget, Christophe Ginevra, Marine Ibranosyan, Philippe Pépin, Amélie Planel, Damien Pognon, Delphine Ponnelle, Alexandra Thabuis, Anne Thuez, Monika Wolska

**Affiliations:** 1 The French Public Health Agency, Auvergne-Rhône-Alpes, Lyon, France; 2 Albertville-Moûtiers Hospital, Albertville, France; 3 Departmental Health Authorities, Savoie, Chambéry, France; 4 Hospices civils de Lyon, National Reference Center of Legionella, Lyon, France; 5 Métropole Savoie Hospital, Chambéry, France; 6 Synlab Laboratory, Albertville, France; 7 Regional Health Authorities, Auvergne-Rhône-Alpes, Clermont-Ferrand, France; 8 Regional Health Authorities, Auvergne-Rhône-Alpes, Lyon, France; 9 The French Public Health Agency, Saint-Maurice, France; 10 The members of the Albertville LD Outbreak Team Investigation are listed under Collaborators

**Keywords:** Legionnaires’ Disease, community outbreak, investigation, Albertville, France

## Abstract

In September 2025, 50 laboratory-confirmed cases of Legionnaires’ disease (LD) were identified in Albertville, in south-eastern France. Initially, 23 patients were only clinically diagnosed and only confirmed later due to limited sensitivity of the urinary antigen screening test used. All cases occurred within 12 days, suggesting a common point source of massive contamination. Despite investigations and the rapid response of LD surveillance partners, the outbreak source has not yet been identified. Vigilance is maintained to detect possible new cases.

On 17 September 2025, clinicians of two hospitals in Savoie, France, reported a total of five patients who had been diagnosed with Legionnaires’ disease (LD) to the local health authorities. As clinicians were witnessing a rapid increase in numbers of patients with signs/symptoms of LD admitted to their hospitals, this suggested an epidemic, so the event and its circumstances were carefully analysed. While the source of the outbreak has not yet been found, we here describe the study conducted so far as well as its results.

## Case definition and epidemiological investigations

A case was defined as a person with pneumonia and laboratory confirmation for *Legionella* with at least one diagnostic test (urinary antigen, PCR, culture), with disease onset from 1 September 2025, and who resided in or had visited the municipality of Albertville (40,000 inhabitants) during the 14 days incubation period.

Cases or their family members were systematically interviewed using a standardised questionnaire to assess potential sources of exposure, individual risk factors, and date of symptom onset. All places visited by the cases during their 14 days incubation period (e.g. home, work, shopping, leisure activities) were geolocated. A heat map highlighting the cases’ residence and their most attended places was created using the geographic information system QGIS [1], in order to define areas for environmental investigations to focus on.

## Description of the outbreak

Between 17 and 27 September 2025, 50 LD cases, residing in or having visited Albertville, were investigated by the local health authorities. LD is a mandatory disease in France and according to the notification data, there was only one LD case linked with Albertville in 2023, which was reported in March, no cases in 2024, and no cases between 1 January and 17 September 2025.

**Figure 1:**
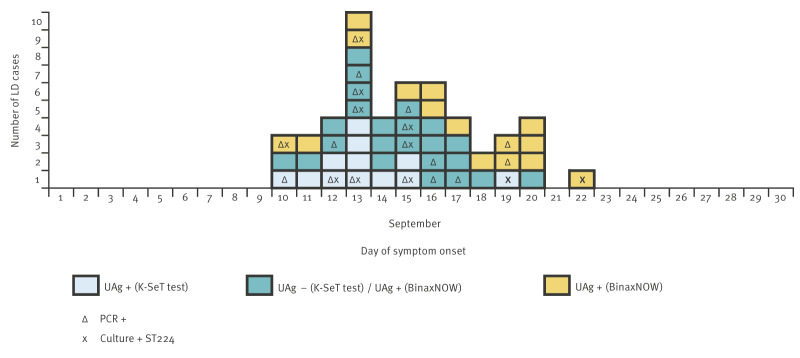
Epidemic curve, diagnostic tests and day of symptom onset of Legionnaires’ disease cases, Albertville, France, September 2025 (n = 50)

LD: Legionnaires’ disease; ST: sequence type; PCR +: testing positive for *Legionella* by PCR; UAg +: testing positive for *Legionella* by urinary antigen; UAg −: testing negative for *Legionella* by urinary antigen.

The epidemic curve shows that the cases occurred over a short period, with onset dates ranging from 10 to 22 September 2025. The number of cases becoming ill reached a peak on 13 September (Figure 1). The shape of the epidemic curve suggests a common point source with an important level of contamination, which started spreading *Legionella* in early September, considering the average incubation period of 2−10 days for LD [2]. Emission from this source was limited in time, given the absence of new cases having onset of illness after 22 September.

The median age of cases was 70 years (range: 34–96 years). The sex ratio (male/female) was 1.3 (28/22). Forty-five cases were hospitalised, including 12 admissions to intensive or continuous care units. Two patients died. Of the five cases that were not hospitalised, three had visited the emergency department and two had consulted their general practitioner. 

At least one risk factor was found in 29 of the 50 cases. The most common risk factors were smoking (n = 17 cases) and diabetes (n = 10 cases).

Among the 50 cases, 39 lived in Albertville and 11, including one person from a Nordic country, reported visiting Albertville during their incubation period (Table).

**Table:** Characteristics of Legionnaires’ disease cases involved in the Albertville outbreak, France, September 2025 (n = 50)

Characteristics	LD cases (n = 50)
	Number
Sex	
Male	28
Female	22
Age (years)	
0–29	0
30–49	7
50–69	18
70–89	20
≥ 90	5
Risk factors	
Smoking	17
Diabetes	10
Other risk factor^a^	14
At least one risk factor	29
Severity	
Hospitalisation	45
- Intensive care unit (ICU)	12
- Death	2
Diagnosis	
Urinary antigen test	50
PCR	18
Culture	11
Residence	
Albertville	39
Surrounding municipalities	5
Other place of residence	6

LD: Legionnaires’ disease.

^a^ Seven of the 14 cases with a risk factor characterised as ‘other’ had pathologies leading to immunosuppression or had immunosuppressive therapy.

Patient interviews failed to identify any shared exposure to public water systems, hotels, whirlpool baths and spas or leisure facilities. Furthermore, investigations did not find any evidence of participation in a particular gathering or other event in early September.

**Figure 2:**
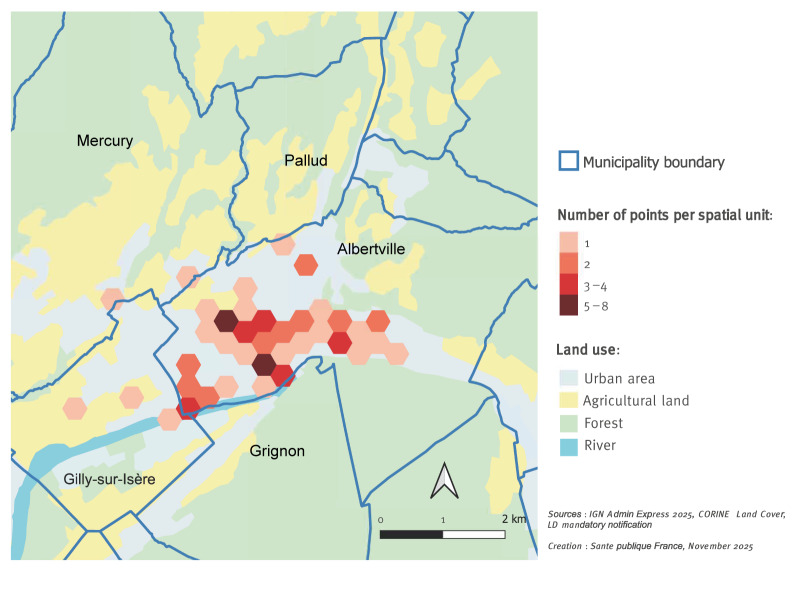
Heat map based on the residence and other places attended by Legionnaires’ disease cases during their incubation period, Albertville, France, September 2025 (n = 50)

The heat map highlights the western part of the municipality of Albertville including commercial areas, with cases spread across the entire urban area (Figure 2).

LD: Legionnaires’ disease.

Points (i.e. hexagons) on the map indicate places that cases lived in or had visited. The colour intensity of each point corresponds to the number of cases that attended the place in question, as indicated by the legend. The time spent at each place was not considered, and each point on the map has the same weight.

## Diagnostic and treatment strategy

Local management of the LD outbreak consisted in alerting hospital and primary care physicians, recommending extensive screening for *Legionella* in all patients with acute respiratory infection from 17 September onwards, followed by the implementation of an empiric treatment strategy as of 18 September using macrolides or fluoroquinolones for all pneumonia patients seen in the emergency department and/or hospitalised. Lower respiratory samples were to be collected from as many cases as possible (even those with symptom onset prior to 18 September) and sent to the National Reference Centre for Legionella in Lyon (NRCL).

Following the hospitalisation of several patients (see below) with a strong clinical suspicion of LD but with a negative urinary antigen test (UAT), their urine samples were sent to the NRCL for additional testing.

This diagnostic and treatment strategy was discontinued on 3 October, after 8 days without any new case.

## Microbiological investigations

All 50 cases had a positive UAT, with either the K-SeT test (Coris BioConcept, Gembloux, Belgium) or BinaxNOW Legionella Urinary Antigen Card, called BinaxNOW test (Abbott, Illinois, United States), depending on the hospital. Respiratory specimen cultures were performed for 35 cases. *Legionella pneumophila* serogroup 1 (Lp1) was isolated from 11 samples, all belonging to sequence type (ST) 224 and harbouring the lag-1 gene (Figure 1). Of the 24 samples with negative cultures, nine tested positive for *L. pneumophila* by PCR. The analysis of results of nested PCR-based sequence-based typing (which allows to determine the ST) from culture-negative, PCR-positive respiratory samples is currently ongoing [3].

**Figure 3:**
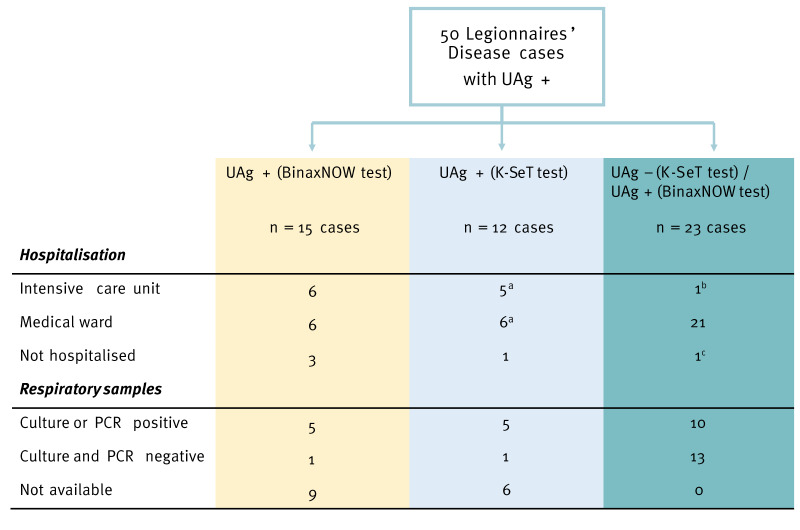
Disease severity level and microbiological results in function of urinary antigen test performed, Albertville, France, September 2025 (n = 50)

Among the 50 cases’ samples, 35 UATs were performed using the K-SeT test and, with this test, 12 samples were positive. Because the 23 patients with a negative UAT using the K-SeT test had a clinical presentation strongly suggestive of LD (all but one were hospitalised with atypical pneumonia), their prior-K-SeT-tested urine samples were re-checked at the NRCL, where they were assayed, after heat treatment, with the BinaxNOW Legionella Urinary Antigen Card. Using this method, each of the 23 samples tested positive for *Legionella* (Figure 3). A respiratory sample was sent to the NRCL for all 23 patients showing initially negative UAT results. Among these, for four patients, PCR was positive for *L. pneumophila*, with culture also positive for Lp1 ST224; moreover, for six patients with negative or no cultures PCR was positive for *L. pneumophila*. Respiratory specimen cultures and PCR were both negative for 13 patients.

UAg +: positive urinary antigen test for *Legionella*; UAg −: negative urinary antigen test for *Legionella*.

^a^ Including one death.

^b^ The case in intensive care unit with UAg- (KseT test) was tested positive in PCR.

^c^ The non-hospitalised case with UAg- (KseT test) was negative in both culture and PCR.

## Environmental investigations and findings

The characteristics of this outbreak with a high number of cases, a sudden onset and geographical dispersion of cases over several kilometres suggest a point source of contamination with significant dispersion capability, such as a cooling tower [4,5]. However, according to environmental authorities, no cooling towers were identified among the industries located in the Albertville area. A wind rose, based on meteorological data recorded in Albertville’s station between 1 and 15 September 2025, shows the predominance of south-westerly winds.

A total of 57 private facilities located in Albertville and the neighbouring Gilly-sur-Isère municipality were contacted by telephone or visited by the local health authorities to identify the presence or absence of equipment with water spray systems that may be a source of airborne contamination. The investigation focused on: wet cooling systems in commercial buildings, irrigation or sprinkler systems, car wash services, etc. At the same time, the municipal authority of Albertville was asked to identify any potentially hazardous installations within the public infrastructures, such as leisure facilities (including an ice rink refrigeration system), public fountains and street cleaners.

The communal biomass heating system was suspected as a potential outbreak source and investigated, by contacting the maintenance company, which provided a description and an operating plan, as this type of facility had already been implicated in an epidemic in France in 2019 [6]. However, this hypothesis was ruled out due to the absence of a condenser. A wastewater treatment plant south-west of places most attended by cases in Albertville was discussed, but taking in account this plant’s distance from the town (ca 5 km) relative to the aerosol spread capacity from activated sludge, it was considered unlikely to be the origin of contamination.

Hot water samples from the houses of 22 cases, apartments of three cases and three residential care facilities for older adults or people with disabilities, were analysed by PCR and culture to verify that their water systems were not contaminated. The results of all sampling activities conducted to date have been negative for Lp1, except for one individual domestic water system.

## Discussion

The LD outbreak in Albertville is the second largest in France (50 cases) since an outbreak in 2003 in northern France (86 cases) [7]. This exceptional situation required the mobilisation and the expertise of various professionals (clinicians, biologists, epidemiologists, engineers and technicians) to conduct epidemiological, microbiological and environmental investigations.

Hospital and private practitioners were advised to be vigilant in their diagnoses, to initiate early treatment for suspected LD cases and to notify the health authorities immediately. Although it is difficult to assess, the early initiation of antibiotic treatment may have prevented more severe cases and deaths. In addition, several press releases were issued to inform the general public of the situation.

Among the 50 cases, all, by definition, presenting with pneumonia, 23 initially tested negative with the K-SeT UAT. Re-analysing their urine samples with another UAT, the BinaxNOW test, yielded a positive *Legionella* diagnostic for all of them. While heating the urine limited the possibility of false positive results with BinaxNOW, this test has prior been shown to have high sensitivity due to the digital reader [8]. The 23 cases were additionally tested through other specimens than urine, including sputum or nasopharyngeal swabs. Using such samples, 10 of the 23 cases further tested positive in culture or PCR. The other 13 cases with negative culture and PCR were also hospitalised for pneumonia in a medical ward, except one who visited the emergency department. A negative PCR or culture from respiratory specimens does not exclude LD when the UAT — performed with validated kits and after heat treatment (which increases specificity) — is positive. Indeed, it has been previously reported that ca 20% of UAT-confirmed Lp1 cases test negative by PCR [9,10]. In our context, PCR and culture negativity in these 23 cases were higher than 20% (13 and 19 cases of 23, respectively) likely reflecting low disease severity, or respiratory specimen quality as sputum and nasopharyngeal swabs were analysed when lower respiratory specimens were not available. While the K-SeT and BinaxNOW tests have been described as having the similar performance for diagnosing LD on concentrated urine samples [7], sensitivity could differ on non-concentrated urine samples and at lower antigen concentrations, particularly for cases with mild symptoms (e.g. one of the 23 cases was not hospitalised and only one of them required admission to the intensive care unit).

It should also be noted that the 23 cases testing negative with the K-SeT all shared the same temporality of illness onset and geographical location as the other LD cases. Thus, altogether, findings suggest that, in the current outbreak, some K-SeT UAT results may have been false negatives. 

The limited sensitivity of the K-SeT UAT [7,8] led to delayed diagnosis of 23 LD cases. Nevertheless, all 23 patients were treated for legionellosis due to highly suggestive clinical presentation and the occurrence of a cluster of cases. This situation highlights the limitations related to sensitivity of diagnostic tests in both non-outbreak and outbreak situations. Beyond the risk of inappropriate antibiotic treatment, delayed diagnosis within a cluster may hinder source identification and delay the implementation of control measures. In this instance, however, no adverse impact from the diagnostic delay was observed, as investigations were initiated from the other confirmed cases. The apparent lower sensitivity of K-SeT test in the context of this outbreak has been reported to the French National Agency for the Safety of Medicines and Health Products (ANSM) and the manufacturer.

The high number of respiratory samples (in 35 of 50 cases) and strains isolated (in 11 of 50 cases) illustrates the effective cooperation of clinicians regarding sample collection.

Microbiological investigations identified a Lp1 strain ST224 as the cause of the outbreak. This genotype is rare in France. Among the 25,778 LD cases recorded in the national database since 2010, an ST was determined for 6,280 cases, including 110 (1.8%) with ST224, reported from various regions across the country but never from the Albertville area. Whole genome sequencing analyses are ongoing to study the relationship between this ST profile and other sporadic cases in France.

The unusually high number of cases within a short period of time and the characterisation of clinical isolates suggest a common source of contamination with high concentrations of Lp1 and substantial potential for spread. Simultaneous contamination of cases through their domestic hot water system seems unlikely as 27 of 28 water samples tested negative. Despite extensive research carried out in Albertville to investigate public and private facilities in the industrial and tertiary sectors, the source has not been identified. Some analysis results are still pending.

The absence of symptomatic cases after 22 September suggests that the outbreak has ended. The emergence of potentially new cases should lead to take in consideration other methods of investigation, such as the use of aerial images to help identify aerosol-generating installations on building roofs [12,13].

While this report is focused on preliminary investigations and their results so far, this study has some limitations. The spatial analysis of the places attended by cases does not take into account the time spent there. Further work could be carried out to potentially refine the delineation of the areas to be investigated. We cannot rule out that official and unofficial communication (through social media, for example) about this outbreak may have led facilities’ managers to implement cleaning and disinfection measures, that may explain the negative results of the sampling campaign. Finally, the presence of aerosol-emitting devices in facilities which are not subject to French regulation were based on self-declaration.

## Conclusion

As the source of contamination has not been identified despite the numerous environmental investigations carried out in Albertville, the possibility of a new outbreak cannot be excluded. It is therefore important to remain vigilant to rapidly detect potentially new cases and implement appropriate diagnostic and treatment strategies. Furthermore, specific investigation methods should be taken into consideration, such as the use of aerial images.

## Data Availability

Data are not publicly available.
